# A genetic analysis identifies a haplotype at adiponectin locus: Association with obesity and type 2 diabetes

**DOI:** 10.1038/s41598-020-59845-z

**Published:** 2020-02-19

**Authors:** Sayantani Pramanik Palit, Roma Patel, Shahnawaz D. Jadeja, Nirali Rathwa, Ankit Mahajan, A. V. Ramachandran, Manoj K. Dhar, Swarkar Sharma, Rasheedunnisa Begum

**Affiliations:** 1 Department of Biochemistry, Faculty of Science, The Maharaja Sayajirao University of Baroda, Vadodara, 390002 Gujarat India; 2 Department of Zoology, Faculty of Science, The Maharaja Sayajirao University of Baroda, Vadodara, 390002 Gujarat India; 3 Human Genetics Research Group, School of Biotechnology, S.M.V.D.U, Katra, 182320 Jammu and Kashmir India; 4 School of Biotechnology, University of Jammu, Jammu, 180001 Jammu and Kashmir India

**Keywords:** Gene expression, Genetic association study

## Abstract

Adiponectin is a prime determinant of the status of insulin resistance. Association studies between adiponectin (*ADIPOQ*) gene single nucleotide polymorphisms (SNPs) and metabolic diseases have been reported earlier. However, results are ambiguous due to apparent contradictions. Hence, we investigated (1) the association between *ADIPOQ* SNPs: −11377C/G, +10211T/G, +45T/G and +276G/T for the risk towards type 2 diabetes (T2D) and, (2) genotype-phenotype association of these SNPs with various biochemical parameters in two cohorts. Genomic DNA of diabetic patients and controls from Gujarat and, Jammu and Kashmir (J&K) were genotyped using PCR-RFLP, TaqMan assay and MassArray. Transcript levels of *ADIPOQ* were assessed in visceral adipose tissue samples, and plasma adiponectin levels were estimated by qPCR and ELISA respectively. Results suggest: (i) reduced HMW adiponectin/total adiponectin ratio in Gujarat patients and its association with +10211T/G and +276G/T, and reduced *ADIPOQ* transcript levels in T2D, (ii) association of the above SNPs with increased FBG, BMI, TG, TC in Gujarat patients and (iii) increased GGTG haplotype in obese patients of Gujarat population and, (iv) association of −11377C/G with T2D in J&K population. Reduced HMW adiponectin, in the backdrop of obesity and *ADIPOQ* genetic variants might alter metabolic profile posing risk towards T2D.

## Introduction

Metabolic Syndrome (MS) is the new wave of diseases that has hit the human population in the last few decades- the Metabolic Syndrome Era. It has become pandemic and with obesity and type 2 diabetes (T2D) clubbed under the MS umbrella, millions of people around the globe have come under its grip. Though obesity and T2D are ubiquitous, there exists a pattern of prevalence based on ethnicity. A recent report has identified demographic transitions, nutrition and lifestyle in the backdrop of genetic predisposition as the chief factors responsible for the rising trend of obesity associated amongst South Asians^1^. Over accumulation of visceral adipose tissue (AT) has been identified as one of the major driving factors towards T2D. Adipose tissue is an important regulator of metabolic homeostasis by virtue of the adipokines (pro-inflammatory and anti-inflammatory) that it secretes. In obese conditions, the fine-tuned balance between the pro- and anti-inflammatory adipokines gets altered leading to various metabolic disorders^2^. These bioactive peptides act locally and distally to calibrate and fine tune various metabolic pathways. Adiponectin is one such calibrator which is abundantly expressed in white adipose tissue^3^. It circulates in three polymorphic forms, low molecular weight (LMW), moderate molecular weight (MMW) and high molecular weight (HMW). Interestingly, the ratio of plasma HMW adiponectin to total adiponectin is more strongly correlated with plasma glucose levels than any of the forms alone^4^. Adiponectin gene (*ADIPOQ/APM1/GBP28*) locus, 3q27, has been strongly associated with a variety of metabolic disorders like- impaired glucose tolerance, obesity, dyslipidemia and T2D^5–7^. Studies undertaken on different ethnic groups have shown positive association of certain SNPs of the adiponectin gene with T2D^3,8–11^. However, T2D being a multi-factorial and polygenic metabolic disorder^12^, significant variations have been reported concerning the genetic architecture underlying T2D amongst different ethnic populations^13,14^. The SNPs to be studied were selected based on the following criteria: (1) validated SNPs for frequency in Genome Wide Association Studies (GWAS), (2) SNPs with scientific evidence for their role in augmented protein synthesis. *ADIPOQ* comprises of 2 introns and 3 exons encoding for the 30 kDa adiponectin protein^15^. Four SNPs were studied, *−*11377C/G (*rs266729*) in promoter, +10211T/G (*rs17846866*) in intron 1, +45T/G (*rs2241766*) in exon 2 and +276G/T (*rs1501299*) in intron 2, to examine their association with T2D. Since Indian population is relatively non-homogenous, we conducted our study in native Gujarat, and Jammu and Kashmir (J&K) population independently. We also aimed to study the genotype-phenotype association of the above-mentioned SNPs with Fasting Blood Glucose (FBG), Body Mass Index (BMI), plasma lipid profile and T2D.

## Materials and Methods

### Study subjects

Two ethnically different populations of India, one from the western Indian state of Gujarat and another from the northern Indian state of J&K were included in the present study. This study was carried out in agreement with the Declaration of Helsinki as approved by the Institutional Ethical Committee for Human Research (IECHR), Faculty of Science, The Maharaja Sayajirao University of Baroda, Vadodara, Gujarat, India (FS/IECHR/2016-9) and Institutional Ethics Review Board (IERB), Shri Mata Vaishno Devi University, Katra, J&K, India (Smvdu/IERB/13/23). It was ensured that at least five previous generations of the study subjects were of the respective ethnicities. Blood collection camps were conducted to guarantee the involvement of all the socio-economic strata in the study. The importance of the study was explained to all the participants and written consent was obtained from all patients, and age and sex-matched control subjects. The study group of Gujarat population included 475 diabetes patients (211 males and 264 females) and 493 control subjects (250 males and 243 females) while, the study group of J&K included 507 diabetes patients (282 males and 225 females) and 300 controls (140 males and 160 females) between the age group of 30 to 67 years. The T2D patients recruited for the study displayed FBG > 125 mg/dL^16^. Patients suffering from autoimmune diseases or cancer were excluded from the study. Samples of visceral (omental) adipose tissue were taken from individuals of Gujarat population undergoing bariatric surgery and fasting clinical parameters of all the study subjects are as described previously^17^. A detailed family history of the patients was recorded based on a questionnaire to collect information on first- and second-degree relatives and their history of T2D. The controls selected showed FBG < 110 mg/dL with no prior history of T2D. They were healthy and disease or infection free. The study subjects included both obese and lean individuals and their BMI (weight in kg/height in m^2^) was calculated by recording height and weight.

### Blood collection and DNA extraction

FBG levels were measured by prick method using glucometer (TRUEresult® - Nipro). Blood was obtained from diabetic and ethnically matched controls as per our previous study^17^. Plasma was used for lipid profiling and assaying plasma HMW adiponectin and total adiponectin levels. PBMCs were separated for DNA extraction by phenol-chloroform method. DNA was stored at −20 °C for further analysis.

### Screening of *ADIPOQ* SNPs

Samples from Gujarat population were genotyped by polymerase chain reaction-restriction fragment length polymorphism (PCR-RFLP) for *−*11377C/G, +10211T/G and +276G/T. The PCR reaction mixture had a total volume of 20 µL as per our previous study^17^ with primer dependent annealing temperatures (Table S1). The amplified products were checked by electrophoresis on a 2.0% agarose gel stained with ethidium bromide. Details of the restriction enzymes (Fermentas, Thermo Fisher Scientific Inc., USA) and digested products are mentioned in Table S1. 15 μl of the amplified products were digested with 1U of the corresponding restriction enzyme in a total reaction volume of 20 μl as per the manufacturer’s instruction. The digestion products with 50 base pair DNA ladder (HiMedia, India) were resolved on 3.5% agarose gels stained with ethidium bromide and visualized under UV transilluminator i.e. E-Gel Imager Life Technologies (Fig. S1A–C) and uncropped images of the gels are as in Fig. S3. More than 10% of the samples were randomly selected for confirmation and the results were 100% concordant (analysis of the chosen samples was repeated by two researchers independently) and further confirmed by sequencing. *ADIPOQ* +45T/ G (*rs2241766*) SNP was genotyped by TaqMan real time PCR using the pre-designed assay ID c__26426077_10 for allelic discrimination, containing specific probes for each allele marked with VIC and FAM fluorescent dyes (ThermoFisher Scientific, USA). Real-time PCR was performed in 10 µl volume using LightCycler^®^480 Probes Master (Roche Diagnostics GmbH, Mannheim, Germany) following the manufacturer’s instructions. A no-template control (NTC) was used with the SNP genotyping assay. Samples with each genotype were analyzed together as an internal control. J&K samples were genotyped for −11377C/G (*rs266729)*, +45T/G *(rs2241766)* and +276G/T*(rs1501299)* in a panel using High-throughput genotyping MassArray platform (SEQUENOM)^18^. The success rate of SNP genotyping was > 95%. As a quality control measure of SNP genotyping, three duplicate samples and a negative control was included in each 96 well plate. The concordance rate for genotyping was 99.5%. Further values for SNP +10211T/G *(rs17846866)* were imputed using CEU data from 1000 genome (Phase 3) as reference dataset and analyzed using PLINK ver 1.07 as the samples were exhausted.

### Plasma parameters

In Gujarat population plasma total cholesterol (TC), triglycerides (TG), and high-density lipoprotein cholesterol (HDL-c) levels were measured using commercial kits (Reckon Diagnostics P. Ltd, Vadodara, India). Low-density lipoprotein cholesterol (LDL-c) was calculated using Friedewald’s (1972) formula^19^. Human total adiponectin and HMW adiponectin ELISA Kits (Elabioscience Biotechnology Inc., USA) with a sensitivity of 0.47 ng/mL and 3.75 ng/mL respectively were used to estimate the levels of total adiponectin and HMW adiponectin in patients and controls. The plasma samples used were freeze-thawed only once. All the plasma estimations were carried out in duplicates with % coefficient of variation within 10%. The plasma samples from J&K population were assayed for various biochemical parameters at a commercial clinical laboratory.

### Determination of adiponectin transcript levels

RNA isolation and cDNA synthesis: Total RNA was isolated from visceral adipose tissue (VAT) using Trizol method. RNA integrity and purity were verified by 1.5% agarose gel electrophoresis/ethidium bromide staining and O.D. 260/280 absorbance ratio of 1.9 respectively. To avoid DNA contamination, RNA was treated with DNase I (Puregene, Genetix Biotech) before cDNA synthesis. Transcriptor High Fidelity cDNA Synthesis Kit (Roche Diagnostic GmbH, Mannheim, Germany) was used to prepare cDNA using one microgram of total RNA isolated, according to the manufacturer’s instructions in the Eppendorf Mastercycler gradient (USA Scientific, Inc., Florida, USA). The expression of *ADIPOQ* and *GAPDH, IPO8* and *ACTB* (reference) transcripts were measured by Light-Cycler^®^ 480 Real-time PCR (Roche Diagnostics GmbH, Manneheim, Germany) using gene- specific primers (Eurofins, Bangalore, India) as shown in Table S1. Real-time PCR was performed using Light-CyclerH 480 SYBR Green I Master (Roche Diagnostics GmbH, Mannheim, Germany) and carried out in the Light-CyclerH 480 Real-Time PCR (Roche Diagnostics GmbH, Mannheim, Germany) as per our previous study^17^.

### Statistical analyses

The normally distributed data for baseline parameters were analyzed by unpaired t-test while Mann-Whitney test was used for data not following normal distribution. Evaluation of the Hardy-Weinberg equilibrium (HWE) was performed for all the SNPs in patients and controls by comparing the observed and expected frequencies of the genotypes using chi-square analysis. The distribution of the genotypes and allele frequencies of *ADIPOQ* SNPs for patients and control subjects were compared using the chi-square test with 2 × 2 contingency tables respectively using GraphPad Prism 5 software. The genotypes have been analyzed in an additive, dominant and recessive model as there was low genotype frequency of the homozygous minor alleles (<10% frequency). *P* values less than 0.0125 for genotype and allele distribution were considered as statistically significant as per Bonferroni’s correction for multiple testing. The strength of association of the *ADIPOQ* SNPs with the risk for T2D was assessed by odds ratio (OR) with 95% confidence intervals (CI). Haplotypes and linkage disequilibrium (LD) coefficients (D′ = D/D_max)_ and r^2^ values for the pair of the most common alleles at each site were obtained using http://analysis.bio-x.cn/myAnalysis.php^20^. Association studies of SNPs with other parameters were performed using analysis of variance (ANOVA) and Kruskal Wallis test. Adjustments for the possible confounding effects of age, sex, and BMI were also done for the samples. Relative gene expression of *ADIPOQ*, and *GAPDH, IPO8* and *ACTB* levels and fold change (2^−∆∆Cp^ values) in T2D patients and control groups were plotted and analyzed by unpaired t-test. All the analyses were carried out in GraphPad Prism 5 software. *P* values less than 0.05 were considered significant for all the association studies. To predict the functional impact of non-coding polymorphisms, ENCODE prediction tool (https://www.encodeproject.org/) was employed^21^.

## Results

Clinical parameters differed significantly between controls and patients in both the populations of Gujarat and J&K (Tables S2 and S3). Patients had significantly higher FBG (*p* < 0.0001). Moreover, obesity related factors like BMI, TC, TG and LDL-c were significantly elevated (*p* < 0.0001, *p* = 0.0360 and *p* = 0.001, respectively) while HDL-c was significantly decreased (*p* < 0.0001) in patients as compared to controls in Gujarat population while in the J&K population BMI (*p* = 0.015), FBG (*p* < 0.0001) and TG (*p* = 0.001) levels were significantly higher in T2D patients.

### Association of *ADIPOQ* SNPs with T2D

The genotype and allele frequencies of the *ADIPOQ* SNPs are summarized in Table 1. The distribution of genotype frequencies for all the polymorphisms investigated was consistent with Hardy-Weinberg Expectations (HWE) (*p* > 0.05) in both the populations. Analysis of the genotype frequencies of +10211T/G (*rs17846866*) and +276G/T (*rs1501299*) SNPs using an additive model revealed them to be significantly associated (*p* < 0.0001) while the promoter 11377C/G (*rs266729*) and exonic +45T/G (*rs2241766*) SNPs were not associated with T2D (Table 1). Further, in Gujarat population a significant association was detected for the intron 1 +10211T/G (*rs17846866*) when analyzed in the recessive model (OR = 1.797, 95% CI = 1.369–2.359, *p* < 0.0001) with T2D. Likewise, the intron 2 + 276G/T (*rs1501299*) SNP was also found to be significantly associated in the recessive model (OR = 2.05, 95% CI, 1.57–2.65, *p* < 0.0001) as shown in Table 1. However, in J&K population, only promoter −11377C/G (*rs266729*) polymorphism was found to be associated (*p* = 0.0101; OR = 1.47, 95% CI = 1.09–1.96) with T2D in the recessive model (Table 1). The frequency of mutant alleles for +10211T/G (*rs17846866*) and +276G/T (*rs1501299*) was noted to be significantly higher in diabetic patients as compared to that of control subjects (OR = 2.33 and OR = 1.726, respectively) in Gujarat population.

**Table 1:** Genotype and allele frequencies distribution of *ADIPOQ* SNPs in T2D patients in Gujarat and J&K population.

SNP	N	Genotype		Allele		Odds Ratio [95% CI] (*p*-value)			
						Allelic	Additive	Dominant	Recessive
**Gujarat Population**									
*rs266729*		CC	CG + GG	C	G	1.23 [0.95–1.59] (0.118)	0.2644	1.46 [0.72–2.95] (0.1443)	1.28 [0.92–1.77] (0.1432)
Controls	286	155	131	427	145				
T2D Patients	285	137	148	402	168				
*rs17846866*		TT	TG + GG	T	G	2.33 [1.85–2.93] (**<**0.0001)	**<**0.0001	1.46 [0.15–2.02] (**<**0.0001)	1.79 [1.36–2.35] (**<**0.0001)
Controls	493	363	130	847	139				
T2D Patients	475	289	186	687	236				
*rs2241766*		TT	TG + GG	T	G	0.86 [0.64–1.18] (0.3722)	0.6704	0.74 [0.22- 2.55] (0.6325)	0.86 [0.61- 1.21] (0.3954)
Controls	467	362	105	822	112				
T2D Patients	359	287	72	642	76				
*rs1501299*		GG	GT + TT	G	T	1.72 [1.42–2.09] (**<**0.0001)	**<**0.0001	1.99 [1.28–3.08] (0.0018)	2.05 [1.57–2.65] (**<**0.0001)
Controls	489	255	216	692	250				
T2D Patients	464	172	298	579	361				
**Jammu and Kashmir Population**									
*rs266729*		CC	CG + GG	C	G	1.34 [1.05–1.69] (0.0168)	0.0365	1.26 [0.67–2.36] (0.2294)	1.47 [1.09–1.96] (0.0101)
Controls	290	151	139	423	157				
T2D Patients	503	309	194	787	219				
*rs17846866*^*#*^		TT	TG + GG	T	G	0.95 [0.70–1.29] (0.3827)	—	—	0.95 [0.71–1.27] (0.3663)
Controls	300	141	159	206	94				
T2D Patients	507	232	275	343	164				
*rs2241766*		TT	TG + GG	T	G	0.72 [0.52–1.02] (0.0613)	0.2041	0.646 [0.23–1.83] (0.2039)	0.71 [0.49–1.04] (0.0788)
Controls	299	251	48	545	53				
T2D Patients	507	400	107	894	120				
*rs1501299*		GG	GT + TT	G	T	1.09 [0.86–1.40] (0.2248)	0.7452	1.12 [0.59–2.13] (0.3670)	1.12 [0.83–1.51] (0.2247)
Controls	289	170	119	443	135				
T2D Patients	502	309	193	786	218				

^#^Values were Imputed using CEU data from 1000 genome (Phase 3) as reference dataset and analyses was carried out in PLINK ver 1.07.

### Haplotype and linkage disequilibrium analysis of *ADIPOQ* SNPs

A haplotype evaluation of four polymorphic sites of *ADIPOQ* was performed in Gujarat population. The estimated frequencies of the haplotypes differed significantly between patients and controls (global *p* = 7.76 × 10^−12^) as shown in Table S4. The disease susceptible haplotypes were CGTG (*p* = 0.0003), CGTT (*p* = 6.32 × 10^−5^), GGTT (*p* = 0.0207) and GGTG (*p* = 0.0030) (Table S4). Furthermore, the GGTG (*p* = 3.87 × 10^−5^) haplotype in particular was found to be significantly higher in obese patients as shown in Table 2. The LD analysis revealed that the four SNPs investigated were in low to moderate LD association (Fig. S2). Haplotype and LD analyses were not performed in the J&K population as only −11377C/G (*rs266729*) was found to be associated with T2D and the genotypes of +10211T/G (*rs17846866*) were imputed.

**Table 2:** Haplotype frequencies in lean and obese patients in Gujarat population.

Haplotype *rs266729, rs17846866, rs2241766, rs1501299*	Obese Patients (Frequency %) (n = 330)	Lean Patients (Frequency %) (n = 150)	*p* for Association	*p* (global)	Odd Ratio [95%CI]
C G T G*	24.49 (0.129)	61.62 (0.081)	0.0397	2.26 × 10^−8^	1.68 [1.020~2.780]
C G T T*	15.12 (0.080)	25.66 (0.034)	0.0053		2.48 [1.285~4.799]
C T G G	12.57 (0.066)	35.80 (0.047)	0.2851		1.43 [0.738~2.791]
C T T G*	53.25 (0.280)	273.96 (0.361)	0.0317		0.67 [0.474~0.968]
C T T T*	17.77 (0.094)	133.56 (0.176)	0.0051		0.47 [0.283~0.809]
G G T G*	15.34 (0.081)	16.02 (0.021)	3.87 × 10^−5^		4.10 [1.993~8.434]
G T T G*	14.89 (0.078)	106.21 (0.140)	0.0219		0.51 [0.293~0.917]
G T T T*	19.89 (0.105)	39.53 (0.052)	0.0072		2.14 [1.215~3.774]

*Indicates haplotypes significantly associated with obesity induced T2D. Frequency **<**0.03 were ignored in the analysis. The haplotypes in J&K population could not be assessed as the data for +10211T/G (*rs17846866*) was imputed.

### *ADIPOQ* expression and plasma HMW adiponectin/total adiponectin ratio in patients and controls

**Figure 1:**
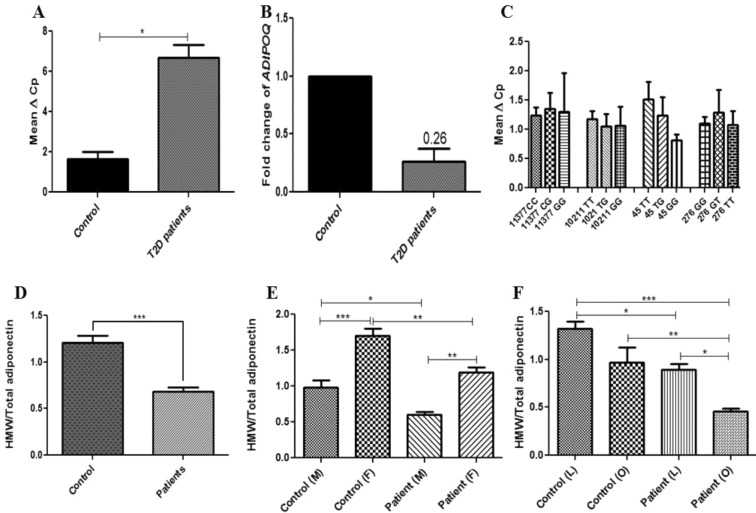
*ADIPOQ* transcript levels and plasma adiponectin levels in Gujarat population. (**A**) Relative gene expression of VAT *ADIPOQ* in controls and patients: Significant decrease in *ADIPOQ* transcript levels was observed in patients (Mean ∆Cp ± SEM: 1.639 ± 0.3829 v/s 6.681 ± 0.6558; *p* = 0.0187), (**B**) Relative fold change of *ADIPOQ* expression in controls and patients. Expression of *ADIPOQ* transcripts in T2D patients as compared to controls was decreased by 0.84 fold as determined by the 2^-ΔΔCp^ method. (Controls n = 14; T2D patients n = 10). (**C**) Association of *ADIPOQ* polymorphisms with *ADIPOQ* transcript levels. No association between *ADIPOQ* polymorphisms and *ADIPOQ* transcript levels (*p* > 0.05). HMW adiponectin/total adiponectin ratio in (**D**) controls versus patients. Plasma HMW adiponectin/total adiponectin ratio in patients were significantly lower than in controls, (**E**) control and diabetic males and females. HMW adiponectin/total adiponectin ratio in control and patient females were significantly higher than in control and patient males and (**F**) lean (L) and obese (O) control and diabetic subjects. Obese patients showed significantly reduced HMW adiponectin/total adiponectin ratio (*p < 0.05, **p < 0.01, ***p < 0.001). (Controls n = 37; T2D patients n = 45).

A significant reduction in *ADIPOQ* transcript levels was observed in Gujarat T2D patients as compared to controls after normalization with *GAPDH* expression (*p* = 0.0187) as suggested by mean ∆Cp values (Fig. 1A). The 2^−ΔΔCp^ analysis showed approximately 0.84 fold decrease in the expression of *ADIPOQ* transcript levels in patients as compared to controls (Fig. 1B). Similar results were obtained for *ADIPOQ* transcript levels when normalized with *IPO8* (*p* = 0.0184) and *ACTB (p* = 0.0344) (Fig. S4A,C). The 2^−ΔΔCp^ analysis of the same showed approximately 0.87 and 0.82 fold reduction in the expression of *ADIPOQ* transcript levels in patients as shown in (Fig. S4B,D). Further, there was no significant difference observed between *ADIPOQ* transcript levels and its SNPs (*p* > 0.05) as shown in Fig. 1C. Plasma HMW adiponectin and total adiponectin levels, and their ratio monitored in 37 controls and 45 patients showed significant decrease (*p* < 0.001) in Gujarat patients as compared to controls (Fig. 1D). Healthy females showed higher HMW adiponectin/total adiponectin ratio than healthy males (*p* < 0.001) (Fig. 1E). A significant drop in the ratio was observed in diabetic males and females when compared with their healthy counterparts (*p* < 0.05 & *p* < 0.01 respectively) (Fig. 1E). There was no significant reduction in the HMW adiponectin/total adiponectin ratio between healthy lean and obese individuals. However, the obese patients showed a significant drop compared to lean patients (*p* < 0.05) (Fig. 1F). Lean and obese diabetic individuals showed reduced HMW adiponectin/total adiponectin ratio as compared to their respective controls (*p* < 0.05, *p* < 0.01). The drop in the plasma adiponectin ratio was further accentuated in obese diabetic patients (*p* < 0.001) (Fig. 1F).

### Association of *ADIPOQ* SNPs and their genotypes with metabolic parameters and HMW adiponectin/total adiponectin ratio

As shown in Table 3, in Gujarat population, the GG genotype of −11377C/G was associated with increased levels of TG, LDL-c and HDL-c (females). The GG genotype of +10211T/G was significantly associated with FBG, BMI, TG, TC, HDL-c and HMW adiponectin/total adiponectin ratio while the TT genotype of +276G/T was significantly associated with increased FBG, BMI, TG, TC and LDL-c and, decreased HDL-c (*p* > 0.05). Further, +45T/G was not associated with any of the parameters in Gujarat population. However, no significant association of the metabolic parameters was observed with the polymorphisms in J&K population (Table S5).

**Table 3:** Genotype-phenotype association analyses of *ADIPOQ* SNPs with metabolic parameters in Gujarat population.

Genotype/Allele	FBG (mg/dL)	BMI (Kg/m^2^)	TG (mg/dL)	TC (mg/dL)	HDL-c (mg/dL)		LDL-c (mg/dL)	HMW adiponectin: total adiponectin (µg/mL)
					Male	Female		
***ADIPOQ*** **−11377 C/G (*rs266729*)**								
**CC**	124.50 (50.02)	25.37 (5.28)	123.00 (79.00)	161.70 (39.47)	36.81 (10.73)	45.17 (14.02)	93.83 (37.5)	0.97 (0.48)
**CG**	124.70 (51.02)	25.57 (5.95)	150.00 (102.00)	162.70 (39.52)	37.59 (9.30)	34.63 (9.96)	101.90 (39.36)	1.00 (0.54)
**GG**	124.10 (30.64)	26.36 (5.51)	166.00 (84.00)	156.40 (37.13)	39.75 (13.25)	26.56 (1.51)	101.40 (32.03)	0.64 (0.24)
***P*** **value**	0.6241	0.4906	**<0.0001**	0.8671	0.7369	**<0.0001**	**0.0087**	0.2055
***ADIPOQ*** +**10211**T**/G (*rs17846866*)**								
**TT**	130.00 (56.13)	25.60 (5.90)	135.80 (92.00)	151.60 (27.89)	42.79 (14.38)	43.18 (14.57)	96.86 (37.5)	1.50 (0.61)
**TG**	132.20 (55.11)	25.33 (5.20)	138.90 (78.00)	162.20 (38.97)	41.62 (21.49)	44.16 (13.51)	96.64 (46.54)	0.86 (0.39)
**GG**	148.10 (56.86)	27.82 (5.60)	166.40 (85.60)	175.60 (39.02)	37.76 (12.92)	34.22 (8.07)	99.20 (37.57)	0.82 (0.36)
***P*** **value**	**<0.0001**	**<0.0001**	**<0.0001**	**<0.0001**	**0.0141**	**<0.0001**	0.6024	**0.0001**
***ADIPOQ*** +**45**T**/G (*rs2241766*)**								
**TT**	155.40 (4.26)	26.82 (5.20)	164.00 (14.8)	163.80 (37.00)	36.62 (11.85)	40.53 (12.36)	95.79 (39.5)	0.98 (1.20)
**TG**	171.50 (12.96)	27.16 (5.29)	172.80 (20.3)	164.50 (44.91)	36.51 (11.00)	40.42 (14.46)	96.75 (39.26)	0.83 (0.38)
**GG**	122.50 (8.50)	30.05 (3.748)	103.90 (15.28)	185.70 (27.61)	34.57 (6.734)	41.27 (11.80)	94.87 (37.83)	0.82 (0.30)
***P*** **value**	0.3293	0.2619	0.6088	0.4735	0.9708	0.9936	0.9396	0.9284
***ADIPOQ*** +**276G/T (*rs1501299*)**								
**GG**	151.00 (53.88)	24.98 (4.53)	143.30 (78.00)	153.20 (29.34)	37.87 (12.34)	40.64 (12.52)	70.36 (27.13)	1.36 (0.63)
**GT**	166.90 (69.67)	27.69 (5.53)	165.20 (89.00)	154.70 (32.12)	35.78 (10.48)	39.25 (12.56)	92.99 (36.33)	0.93 (0.44)
**TT**	303.80 (94.54)	29.75 (4.23)	266.60 (90.00)	189.00 (25.96)	33.28 (11.93)	37.34 (6.34)	90.62 (34.1)	0.75 (0.33)
***P*** **value**	**<0.0001**	**0.0001**	**<0.0001**	**0.0001**	**<0.0001**	0.0831	**0.005**	**0.0006**

Data represented as Mean (SD).

### Bioinformatics analyses

ENCODE data base showed that −11377C/G (*rs266729*), +10211T/G (*rs17846866*), +45T/G (*rs2241766*) and +276G/T (*rs1501299*) do not overlap with any cis-Response Elements (cREs) or display any cREs within 2 kb. Further, eQTL database GTex shows TG and GG genotypes of *rs17846866* to have significantly reduced levels of plasma adiponectin similar to our findings. However, the eQTL data for the rest of the SNPs are not available. Analysis of *rs2241766*, a synonymous exonic SNP, revealed that the glycine residue at the 15th position remains unchanged (SIFT). Further, the change in codon usage was calculated by applying a relative synonymous codon usage (RSCU) approach to understand the relevance of ribosomal pause in reduced amount of protein being expressed. The delta Relative Synonymous Codon Usage (RSCU) value for the GGT to GGG codon change was calculated to be −0.31. However, no significant association of the +45T/G polymorphism was found with adiponectin levels.

## Discussion

Our findings, for the first time, collectively suggest that *ADIPOQ* CGTG, CGTT, GGTT and GGTG haplotypes were associated with T2D, further GGTG was significantly associated with obesity induced T2D. Also, +10211T/G (*rs17846866*) and +276G/T (*rs1501299*) were strongly associated with obesity induced T2D susceptibility in Gujarat population; whereas in J&K population only −11377C/G (*rs266729*) was found to be associated with T2D. The difference in the association of variants can be attributed to the ethnic differences between the two populations. The findings in Gujarat population are further linked with reduced levels of HMW adiponectin and disease-associated risk factors like FBG, BMI and lipid parameters thereby suggesting their crucial role in metabolic disease susceptibility.

Obese phenotype has been associated with a reduction in the anti-inflammatory and a boost in the pro-inflammatory adipokines. Our previous reports suggest interleukin 1β (IL1β)^22^, resistin^23^ and TNFα^24^ to play an important role in the development of obesity, islet dysfunction and decreased insulin secretion. On the contrary, adiponectin^2^, omentin-1^25^, melatonin^26^ and vaspin^27^ are known to enhance insulin sensitivity. The normal range of total adiponectin in healthy individuals is reported to be 2–20 µg/mL^28^. The characteristic short stature of South Asians combined with visceral adiposity leads to an increased weight per area distribution defined by body mass index predisposing those to metabolic diseases^1,29–31^. Genome-wide association studies have shown a close association between adiponectin, *ADIPOQ* SNPs, fasting hyperglycemia and various metabolic diseases though varying from population to population^32–34^. Earlier studies have shown promoter −11377C/G (*rs266729*) polymorphism to have a positive association with hypoadiponectinemia and risk of developing T2D^35^ and is supported by the findings in J&K population. As opposed to this, we found this SNP not to be associated with T2D or BMI in Gujarat population supporting the work by Schaffler *et al*. who also reported the absence of transcription factor binding sites at or around this SNP site^36^. However, the GG genotype of −11377 C/G (*rs266729*) did show an association with increased serum triglycerides and LDL-c, and reduced HDL-c in females. In spite of not being associated with T2D, possibly an indirect effect of other SNPs could be the reason for the observed altered association of the −11377 C/G (*rs266729*) with the serum lipid levels.

**Figure 2:**
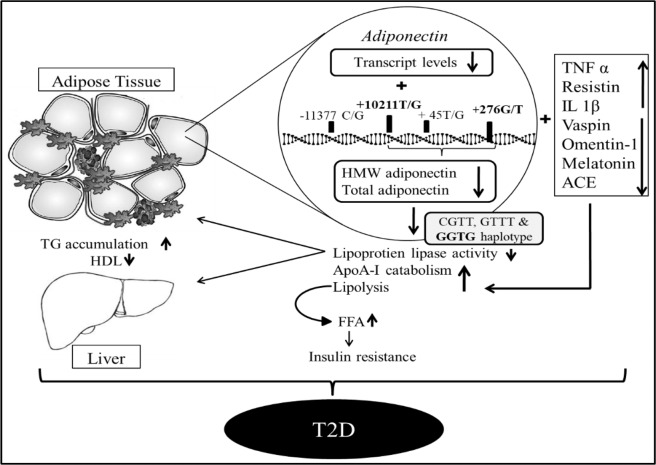
Role of *ADIPOQ* SNPs in T2D: The *ADIPOQ* CGTT, GTTT and GGTG haplotypes in presence of *ADIPOQ* +10211T/G (*rs17846866*) and +276G/T (*rs1501299*) along with decreased transcript, plasma HMW adiponectin and total adiponectin, and increased TNFα, FFA, resistin leads to altered metabolic profile thereby contributing to insulin resistance and T2D in Gujarat population.

Adiponectin gene expression in an adipose tissue is regulated by a 34 bp enhancer located in the first intron^37^. Therefore, the finding of +10211T/G (*rs17846866*) located close to the enhancer in the region of the first intron affecting lipid metabolism and adiponectin levels in the present study is of significance. Though the ENCODE data base doesn’t show an overlap of this polymorphism with any cREs or display any cREs within 2 kb; eQTL database GTex shows TG and GG genotypes of +10211T/G (*rs17846866*) to have significantly reduced levels of plasma adiponectin similar to our findings. Additionally, this SNP is also seen to be associated with increased BMI, FBG, TG, TC and reduced HDL-c. To date, three independent studies, including ours, have established the association of +10211T/G (*rs17846866*) with three different Indian populations belonging to different demographical and geographical regions, thus further validating the significance of this SNP^10,11^. However, the results from J&K population did not reveal any such association. +45T/G (*rs2241766*) is a synonymous SNP with a codon change from GGT to GGG. Though studies on Chinese Han population found an association between +45T/G (*rs2241766*) and insulin resistance^38^; our results show no association between +45T/G (*rs2241766*) and T2D as supported by studies on Italian, French and Swedish populations^3,8,9^. We report a significant association of +276G/T (*rs1501299*) with T2D, and serum lipid profile in Gujarat population while no association was found in J&K population. Supporting our data from Gujarat population, similar results were obtained in earlier studies in German^39^, Swedish^40^, Italian Caucasian^41^, French Caucasian^3^ and South Indian populations^35^. However, the results of the study by Hara *et al*.^42^ in Japanese subjects were in accordance with the results obtained in J&K population. In Gujarat population, the TT genotype conferred approximately double risk for developing T2D against the GG genotype in +276G/T (*rs1501299*). Furthermore, +276G/T (*rs1501299*) is also found to be linked with increased BMI, FBG, TG, and TC, and reduced HDL-c in males. These findings also suggest the association of +276G/T (*rs1501299*) with Non-Alcoholic Fatty Liver Disease (NAFLD), co-morbidity associated with T2D as supported by Wang *et al*.^43^. Additionally, we have also found increased levels of TNFα, Free Fatty Acids (FFA) and resistin in obese patients^17,44^. Since TNFα is shown to be an important regulator of adiponectin multimerization^45^, our observations of increased TNFα, reduced adiponectin transcript and HMW adiponectin levels in obese patients are self-explanatory. We had also reported a rise in IL1β levels in obese diabetic patients^46^, asserting the rise in pro-inflammatory adipokine and drop in anti-inflammatory adipokine in obesity-associated low-grade inflammatory condition. Further, adiponectin levels show sexual dimorphism^47^ and our results further confirm this as females in general demonstrated a higher tendency of HMW adiponectin/total adiponectin ratio than males. Also, a significant drop in adiponectin ratio of lean diabetic individuals was observed which was further pronounced in obese diabetic patients. Moreover, the overall plasma HMW adiponectin/total adiponectin ratio tends to be lower in subjects with the homozygous mutant allele for +10211T/G (*rs17846866*) and +276G/T (*rs1501299*). In concordance with our findings, adiponectin levels were strongly and inversely associated with diabetes risk^48,49^. Alongside, we had also reported the prevalence of a significantly high number of angiotensin convertase enzyme (ACE) I/D polymorphism in the same population^50^. The ACE D allele has in particular been shown to be associated with increased angiotensin II^51^ which may be further adding to the down regulation of adiponectin. We suggest that the reduced HMW adiponectin in particular is responsible for insulin resistance as, among the adiponectin isoforms, the HMW isoform binds to its receptor with maximum affinity leading to a potent activation of 5′ AMP-activated protein kinase (AMPK). Thus, the lowered HMW adiponectin may be partly responsible for developing T2D^52^. The increased level of TG may be due to a decrease in the lipoprotein lipase activity and Very Low-Density Lipoprotein receptor (VLDLr) expression levels, which have been proposed to be modulated by adiponectin^53^. While HDL-c levels and their particle size are inversely correlated with the catabolic rate of apolipoprotein (ApoA-I), a direct role of reduced adiponectin with increased catabolism of the major ApoA-I present in HDL-c has been proposed^54^, explaining how hypoadiponectinemia leads to decreased HDL-c levels. The correlation between hypoadiponectinemia and reduced HDL-c levels, as observed by us further strengthens the hypothesis. To summarize, +10211T/G (*rs17846866*) and +276G/T (*rs1501299*) are significantly associated with increased FBG, BMI, TG, TC and reduced HMW adiponectin/total adiponectin ratio. More importantly, the haplotype analysis reveals that individuals with GGTG haplotype in particular show an increased tendency towards obesity induced T2D^55^ (Fig. 2). Thus, we may conclude that adiponectin gene is associated with T2D, nonetheless variation in the susceptibility loci within the gene depends on ethnic variation among different populations. However, further investigations to understand the mechanistic aspects of genetic variants regulating adiponectin levels are warranted in other cohorts.

## Supplementary information

Supplementary data.

## Data Availability

The datasets generated during and/or analyzed during the current study are available from the corresponding author on reasonable request.
