# Publisher Correction: A genetic analysis identifies a haplotype at adiponectin locus: Association with obesity and type 2 diabetes

**DOI:** 10.1038/s41598-020-62762-w

**Published:** 2020-04-27

**Authors:** Sayantani Pramanik Palit, Roma Patel, Shahnawaz D. Jadeja, Nirali Rathwa, Ankit Mahajan, A. V. Ramachandran, Manoj K. Dhar, Swarkar Sharma, Rasheedunnisa Begum

**Affiliations:** 1 Department of Biochemistry, Faculty of Science, The Maharaja Sayajirao University of Baroda, Vadodara, 390002 Gujarat India; 2 Department of Zoology, Faculty of Science, The Maharaja Sayajirao University of Baroda, Vadodara, 390002 Gujarat India; 3 Human Genetics Research Group, School of Biotechnology, S.M.V.D.U, Katra, 182320 Jammu and Kashmir India; 4 School of Biotechnology, University of Jammu, Jammu, 180001 Jammu and Kashmir India

Correction to: *Scientific Reports* 10.1038/s41598-020-59845-z, published online 19 February 2020

In the original version of this Article, the ORCID ID for Rasheedunnisa Begum was omitted. This has now been corrected in the HTML and PDF versions of the Article.

