# Supplementary material for: A genetic analysis identifies a haplotype at adiponectin locus: Association with obesity and type 2 diabetes
Source: Sci Rep. 2020 Feb 19;10:2904. doi: 10.1038/s41598-020-59845-z (PMC7031532; doi:10.1038/s41598-020-59845-z)
Supplement: Supplementary file 1 — Supplementary data. [file 41598_2020_59845_MOESM1_ESM.docx]

# A genetic analysis identifies a haplotype at adiponectin locus: Association with obesity and type 2diabetes

Sayantani Pramanik Palit^1^, Roma Patel^1#^, Shahnawaz D Jadeja^1#^, Nirali Rathwa^1^, Ankit Mahajan^3,4^, A.V. Ramachandran^2^, Manoj K. Dhar ^4^, Swarkar Sharma ^3^, RasheedunnisaBegum^1^*

1 Department of Biochemistry, Faculty of Science, The Maharaja Sayajirao University of Baroda,

Vadodara-390002, Gujarat, India;

2 Department of Zoology, Faculty of Science, The Maharaja Sayajirao University of Baroda, Vadodara- 390002, Gujarat, India;

^3^Human Genetics Research Group, School of Biotechnology, S.M.V.D.U, Katra, Jammu and Kashmir, India 182320;

^4^School of Biotechnology, University of Jammu, Jammu, Jammu and Kashmir, India 180001.

^#^ Contributed equally to the work.

*** Corresponding author:**

Department of Biochemistry,

Faculty of Science,

The Maharaja Sayajirao University of Baroda,

Vadodara- 390002, Gujarat, India;

E-mail: [rasheedunnisab@yahoo.co.in](mailto:rasheedunnisab@yahoo.co.in)

Tel: +91-265-2795594

**Table S1. Details of primers and restriction enzymes used in the study**

| **Gene/SNP** | **Primer sequence**  **(5’ to 3’)** | **Annealing Temperature (^0^C)** | **Amplicon**  **Size**  **(bp)** | **Restriction Enzyme** | **Digested products**  **(bp)** |
| --- | --- | --- | --- | --- | --- |
| **(rs266729)**  -11377 C/G FP  -11377 C/G RP | GCTCTGTGTGGACTGTGGAG  TAGAAGCAGCCTGGAGAACT | 61.3 | 303 | *Hha*I | 181 bp +122 bp |
| **(rs17846866)**  +10211T/G FP  +10211T/G RP | GCTAAGTATTACAGATTTCAGGGCAG  CAGCCATGGAGAGACAGACCC | 62 | 293 | *Hinf*I | 132bp + 107bp + 54bp |
| **(rs1501299)**  +276 G/T FP  +276 G/T RP | GATGCAGCAAAGCCAAAGTC  TGGCCTCTTTCATCACAGACC | 61 | 196 | *Bsm*I | 148 bp + 48 bp |
| ***ADIPOQ***  FP  RP | AACATGCCCATTCGCTTTACC  GACTGTGATGTGGTAGGCAA | 67 | 107 | NA | NA |
| ***IPO 8***  FP  RP | CGAGCTCAACCAGTCCTACA  TCTGGCCAGTATTGTGTCACC | 68 | 139 | NA | NA |
| ***ACTB***  FP  RP | ACTCTTCCAGCCTTCCTTCC  CGTACAGGTCTTTGCGGATG | 67 | 104 | NA | NA |
| ***GAPDH***  FP  RP | CATCACCATCTTCCAGGAGCGAG  CCTGCAAATGAGCCCCAGCCT | 65 | 122 | NA | NA |

FP-Forward Primer, RP- Reverse Primer, bp- base pairs

**Table S2. Baseline characteristics of controls and patients from Gujarat population.**

|  | **Controls**  Mean ± SD |  | **Patients**  Mean ± SD | **P value** |
| --- | --- | --- | --- | --- |
| Age (years)  Sex: Male  Female  FBG (mg/dL)  BMI (Kg/m^2^)  TC (mg/dL)  TG (mg/dL)  HDL cholesterol (mg/dL)  LDL cholesterol (mg/dL)  Onset age (years)  Duration of disease (years) | (n =493) |  | (n =475) |  |
|  | 49.64 ± 16.35  250 (52.6%)  243 (51.2%)  100.1 ± 7.32  24.24 ± 5.2  160.9 ± 22.2  111.7 ± 25.90  42.79 ± 15.94  84.69 ± 28.06  NA  NA |  | 55.99 ± 10.42  211 (44.5%)  264 (55.5%)  155.3 ± 32.09  27.04 ± 5.1  166.2 ± 19.68  164.5 ± 11.1  38.2 ± 12.6  110.7 ± 29.2  50.65 ± 10.10  8.06 ± 7.3 | -  -  -  <0.0001  <0.0001  0.036  0.001  <0.0001  <0.0001  -  - |

**Table S3. Baseline characteristics of controls and patients from J&K population.**

|  | **Controls** |  | **Patients** | **P value** |
| --- | --- | --- | --- | --- |
|  | Mean ± SD |  | Mean ± SD |  |
|  | (n=300) |  | (n=507) |  |
| Age (years) | 44.53 ± 10.95 |  | 50.04 ± 9.74 | - |
| Sex: Male | 140 (46.6%)  160 (53.3%) |  | 282 (55.6%)  225 (44.3%) |  |
| Female |  |  |  |  |
| BMI (Kg/m^2^ ) | 25.7±4.2 |  | 26.5±4.8 | 0.015 |
| FBG (mg/dl) | 82.0±7.8 |  | 158.3±56.3 | <0.0001 |
| TC (mg/dl) | 169.5±21.5 |  | 172.6±29.9 | 0.120 |
| TG (mg/dl) | 180.3±69.1 |  | 206.4±105.7 | 0.001 |
| HDL cholesterol (mg/dl) | 169.5±21.5 |  | 172.6±29.9 | 0.120 |
| LDL cholesterol (mg/dl) | 82.7±18.6 |  | 82.4±23.9 | 0.870 |
|  |  |  |  |  |

**Table S4. Haplotype frequencies in T2D patients and controls in Gujarat population.**

| **Haplotype**  ***rs 266729, rs17846866, rs2241766,***  ***rs1501299*** | **Patients**  **(Frequency %)**  **(n=494)** | **Controls**  **(Frequency %)**  **(n=458)** | ***p***  **for Association** | ***p* (global)** | **Odd Ratio**  **[95%CI]** |
| --- | --- | --- | --- | --- | --- |
| **C G T G*** | 61.86(0.12) | 26.32(0.05) | 0.0003 | 7.76 x10^-12^ | 2.37 [1.476~3.829] |
| **C G T T*** | 32.98(0.06) | 6.90(0.015) | 6.32 x 10^-5^ |  | 4.72 [2.058~10.849] |
| C T G G | 18.31(0.03) | 26.38(0.05) | 0.1398 |  | 0.63 [0.344~1.167] |
| C T T G | 135.16(0.27) | 200.65(0.43) | 1.03x10^-7^ |  | 0.47 [0.361~0.626] |
| C T T T | 88.43(0.17) | 66.34(0.14) | 0.1390 |  | 1.30 [0.917~1.846] |
| **G G T G*** | 21.95(0.04) | 5.66(0.01) | 0.0030 |  | 3.74 [1.474~9.534] |
| **G G T T*** | 16.72(0.03) | 5.25(0.01) | 0.0207 |  | 3.04 [1.132~8.188] |
| G T T G | 51.96(0.10) | 71.78(0.15) | 0.0195 |  | 0.63 [0.432~0.931] |
| G T T T | 34.95(0.07) | 22.09(0.04) | 0.1362 |  | 1.51 [0.874~2.625] |

*Indicates haplotypes significantly associated with T2D. Frequency<0.03 were ignored in the analysis.

The haplotypes in J&K population could not be assessed as the data for +10211T/G (*rs17846866)* was imputed.

# Table S5. Genotype-phenotype association analyses of *ADIPOQ* SNPs with metabolic parameters in J&K population.

| **Genotype/ Allele** | **FBG** | **BMI** | **TG** | **TC** | **HDL-c (mg/dL)** | | **LDL-c** |
| --- | --- | --- | --- | --- | --- | --- | --- |
|  | **(mg/dL)** | **(Kg/m2)** | **(mg/dL)** | **(mg/dL)** | **Male** | **Female** | **(mg/dL)** |
| ***ADIPOQ* -11377 C/G (*rs266729*)** | | | | | | | |
| **CC** | 135.42 (63.25) | 26.19 (4.78) | 203.68 (72.07) | 170.77 (24.52) | 46.09 (12.08) | 51.49 (6.17) | 81.54 (22.72) |
| **CG** | 123.4 (52.24) | 26.27 (4.88) | 187.43 (72.24) | 171.41 (27.36) | 46.04 (4.85) | 51.21 (9.8) | 83.85 (21.83) |
| **GG** | 128.75 (61.71) | 26.76 (3.7) | 211.55 (115.81) | 172.68 (22.91) | 45.51 (4.04) | 50.68 (4.14) | 79.47 (18.73) |
| ***P* value** | 0.27 | 0.75 | 0.1 | 0.88 | 0.9 | 0.89 | 0.35 |
| ***ADIPOQ* +45 T/G *(rs2241766)*** | | | | | | | |
| **TT** | 130.02 (60.14) | 26.19 (4.8) | 200.52 (95.5) | 171.25 (26.09) | 46.26 (10.48) | 51.27 (7.78) | 81.98 (22.59) |
| **TG** | 133.26 (58.6) | 26.34 (4.7) | 187.67 (113.8) | 170.69 (22.6) | 45.40 (4.08) | 51.52 (6.82) | 83.58 (20.83) |
| **GG** | 131.44 (55.14) | 27.69 (2.70) | 188.34 (52.50) | 162.57 (26.47) | 45.96 (2.31) | 51.39 (7.59) | 76.71 (15.19) |
| ***P* value** | 0.84 | 0.42 | 0.42 | 0.42 | 0.82 | 0.4 | 0.56 |
| ***ADIPOQ* +276 G/T *(rs1501299)*** | | | | | | | |
| **GG** | 131.56 (59.92) | 26.51 (4.87) | 193.63 (91.95) | 169.56 (24.27) | 45.75 (4.00) | 50.92 (5.30) | 81.91 (22.87) |
| **GT** | 131.52 (60.46) | 25.99 (4.66) | 205.76 (111.37) | 173.84 (27.9) | 46.87 (16.03) | 51.69 (10.38) | 82.97 (21.98) |
| **TT** | 120.03 (51.54) | 25.19 (4.05) | 201.28 (89.15) | 169.04 (22.41) | 45.42 (9.68) | 52.95 (6.5) | 79.88 (16.44) |
| ***P* value** | 0.48 | 0.12 | 0.34 | 0.12 | 0.5 | 0.47 | 0.72 |

# Data represented as Mean (SD).

**
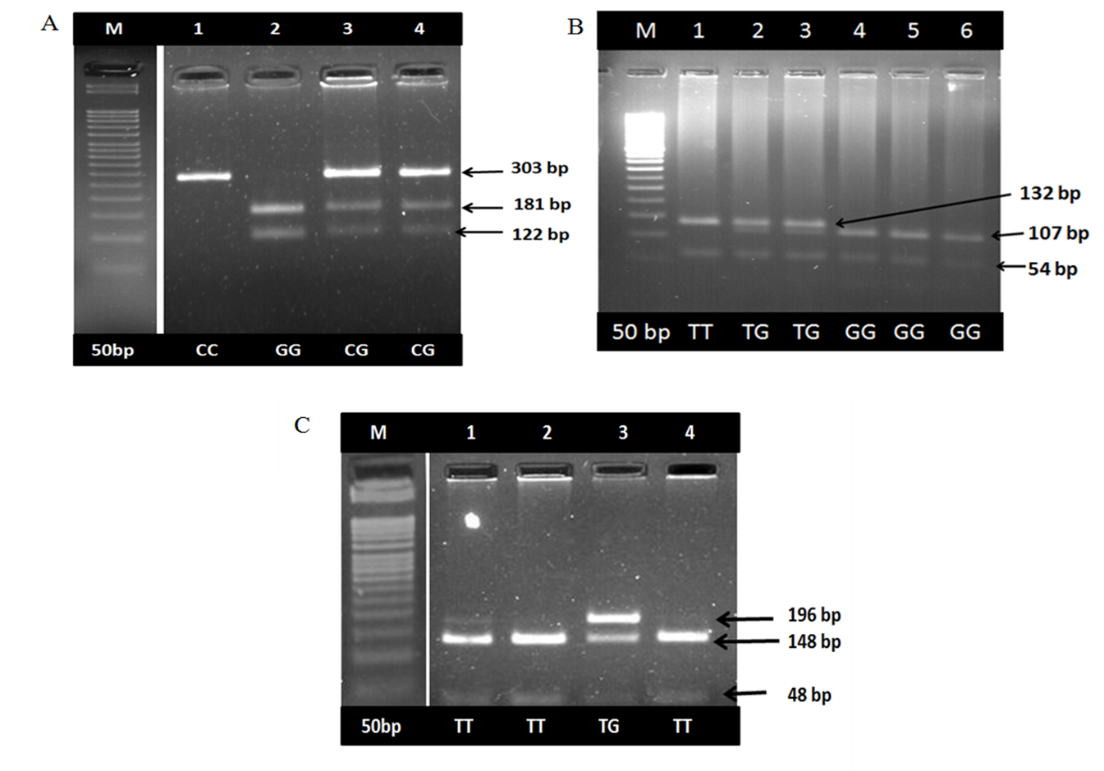
**

**Figure S1: PCR-RFLP analysis of *ADIPOQ* -11377** (*rs266729*) **C/G, +10211 T/G** (*rs17846866*), and **+276 G/T** (*rs1501299*)**:** A) PCR-RFLP analysis of -11377C/G (*rs266729*) on 3.5% agarose gel electrophoresis; lane 1: shows homozygous (CC) genotype, lane 2: shows homozygous (GG) genotype; lane 3 & 4: shows heterozygous (CG) genotype. B) PCR-RFLP analysis of +10211 T/G (*rs17846866)* on 3.5% agarose gel electrophoresis; lane 1: shows homozygous (TT) genotype, lanes 2 & 3: shows homozygous (TG) genotype; lane 4, 5 & 6: shows homozygous (GG) genotype. C) PCR-RFLP analysis of +276 T/G (*rs1501299*) on 3.5% agarose gel electrophoresis; lane 1, 2 & 4: shows homozygous (TT) genotype, lanes 3: shows heterozygous (TG) genotype. Gel images were cropped and merged (demarcated with white space) with marker to give a precise view of the obtained genotypes.


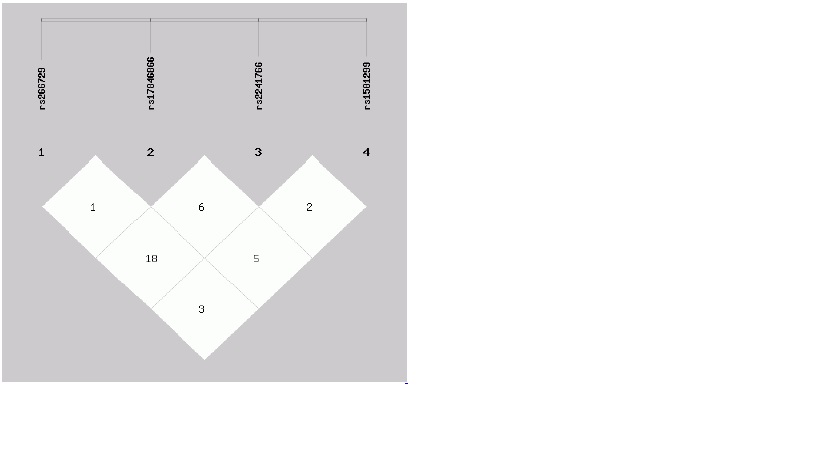


**Figure S 2: Linkage disequilibrium analysis of** ***ADIPOQ* SNPs in Gujarat population.** LD block of *ADIPOQ* -11377 C/G (*rs266729*), +10211 T/G *(rs17846866)*, +45 T/G *(rs2241766)* and +276 G/T *(rs1501299)* SNPs show low to moderate LD association. LD association analysis of the SNPs in J&K population could not be assessed as the data for +10211T/G (*rs17846866)* was imputed.


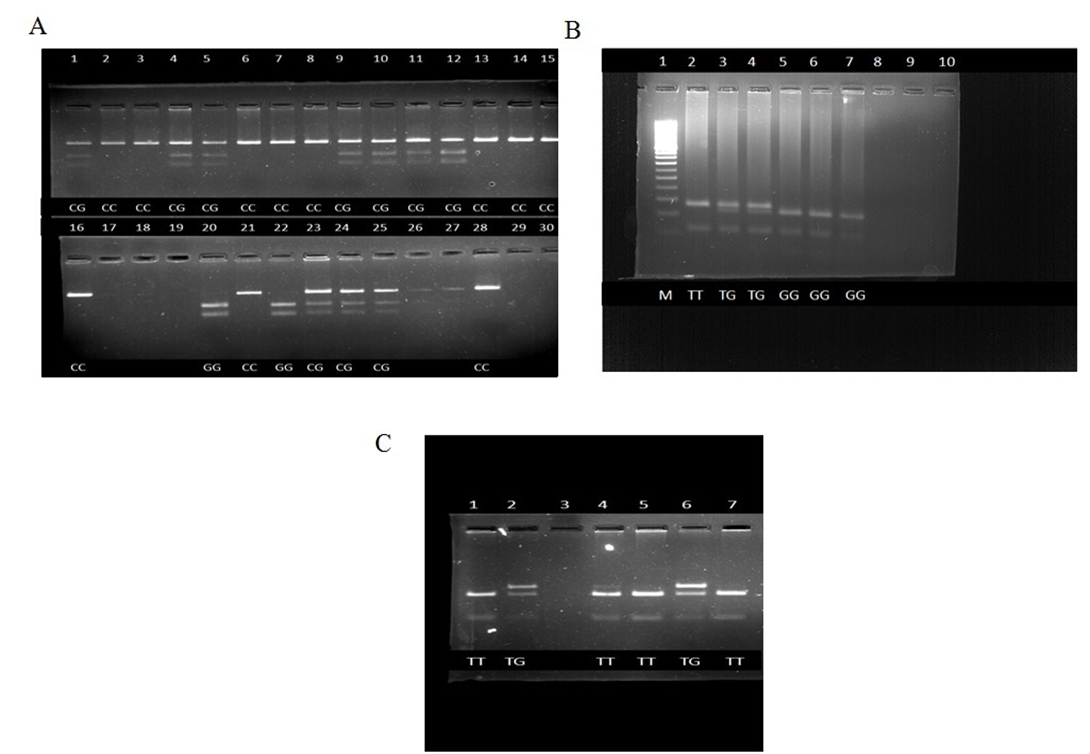


**Figure S3: Uncropped images of PCR-RFLP analysis of *ADIPOQ* -11377** (*rs266729*) **C/G, +10211 T/G** (*rs17846866*)**, +45 T/G** (*rs2241766*) **and +276 G/T** (*rs1501299*)**:** A) PCR-RFLP analysis of -11377C/G (*rs266729*) on 3.5% agarose gel electrophoresis; lane 2, 3, 6, 7, 8, 13, 14, 15, 16, 21 & 28: shows homozygous (CC) genotype, lane 20 & 22: shows homozygous (GG) genotype; lane 1, 4, 5, 9, 10, 11, 12, 23, 24 & 25: shows heterozygous (CG) genotype. B) PCR-RFLP analysis of +10211 T/G (*rs17846866)* on 3.5% agarose gel electrophoresis; lane 1: shows 50bp ladder, lane 2: shows homozygous (TT) genotype, lanes 3 & 4: shows homozygous (TG) genotype; lane 4, 5 & 6: shows homozygous (GG) genotype. C) PCR-RFLP analysis of +276 T/G (*rs1501299*) on 3.5% agarose gel electrophoresis; lane 1, 2 & 4: shows homozygous (TT) genotype, lanes 3: shows heterozygous (TG) genotype. Wells without genotype were repeated.


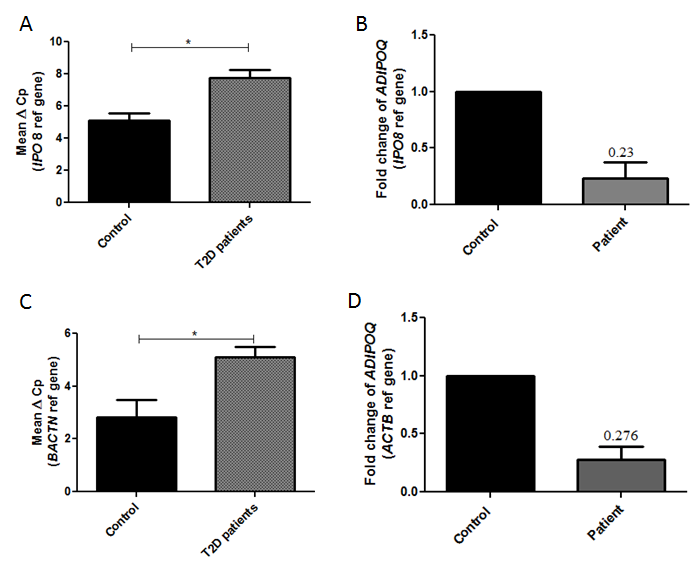


**Figure S4. *ADIPOQ* transcript levels in Gujarat population. A) Relative gene expression of VAT *ADIPOQ/ IPO8* in controls and patients:** Significant decrease in *ADIPOQ* transcript levels was observed in patients as compared to controls (Mean ∆Cp ± SEM: 5.12 ± 0.45 v/s 7.766 ± 0.49; p=0.0163). **B) Relative fold change of *ADIPOQ/IPO8* expression in controls and patients.** Expression of *ADIPOQ* transcripts in T2Dpatients as compared to controls was decreased by 0.87 fold as determined by the 2^-ΔΔCp^ method. **C) Relative gene expression of VAT *ADIPOQ/ACTB* in controls and patients:** Significant decrease in *ADIPOQ* transcript levels was observed in patients (Mean ∆Cp ± SEM: 2.84 ± 0.63 v/s 5.11 ± 0.38; p=0.0378). **D) Relative fold change of *ADIPOQ/ACTB* expression in controls and patients.** Expression of *ADIPOQ* transcripts in T2D patients as compared to controls was decreased by 0.82 fold as determined by the 2^-ΔΔCp^ method (Controls n=14; T2D patients n=10).
